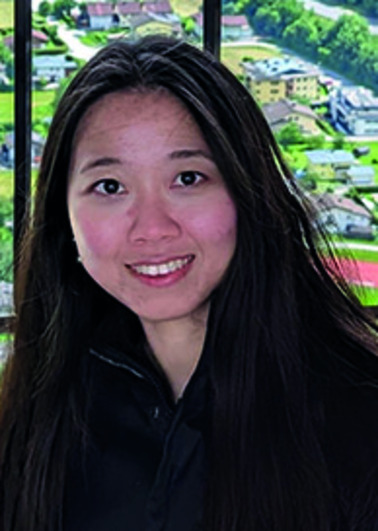# Exploring Aspartate Transcarbamoylase: A Promising Broad‐Spectrum Target for Drug Development

**DOI:** 10.1002/cbic.202401009

**Published:** 2025-03-27

**Authors:** Siyao Chen, Queenie Mondile, XiaoChen Du, Chao Wang, Mayur Mukim, Carsten Wrenger, Alexander S. S. Dömling, Özlem Tastan Bishop, Matthew R. Groves

**Affiliations:** ^1^ Department of Chemical and Pharmaceutical Biology University of Groningen Antonius Deusinglaan 1 9731AV Groningen The Netherlands; ^2^ Research Unit in Bioinformatics (RUBi) Department of Biochemistry Microbiology and Biochemistry Rhodes University; ^3^ Neurobiology MRC-Laboratory of Molecular Biology Cambridge Biomedical Campus Francis Crick Ave, Trumpington Cambridge CB2 0QH; ^4^ Unit for Drug Discovery Department of Parasitology Institute of Biomedical Sciences University of São Paulo Avenida Professor Lineu Prestes 1374 05508-000 São Paulo-SP Brazil; ^5^ Czech Advanced Technology and Research Institute (CATRIN) and Institute of Molecular and Translational Medicine (IMTM Faculty of Medicine and Dentistry Palacky University Šlechtitelů 27 779 00 Olomouc Czech Republic; ^6^ National Institute for Theoretical and Computational Sciences (NITheCS) South Africa; ^7^ Genomics for Health in Africa (GHA) Africa-Europe Cluster of Research Excellence (CoRE)

**Keywords:** *de novo* Pyrimidine Biosynthesis, Aspartate Transcarbamoylase, Allosteric Inhibition, Infectious diseases, Broad spectrum drug discover

## Abstract

Pyrimidine nucleotides are essential for a wide variety of cellular processes and are synthesized either via a salvage pathway or through *de novo* biosynthesis. The latter is particularly important in proliferating cells, such as infectious diseases and cancer cells. Aspartate transcarbamoylase (ATCase) catalyzes the first committed and rate‐limiting step in the *de novo* pyrimidine biosynthesis pathway, making it an attractive therapeutic target for various diseases. This review summarizes the development of a series of allosteric ATCase inhibitors, advancing them as potential candidates for malarial, tuberculosis and cancer therapies. Furthermore, it explores the potential for these compounds to be expanded into drugs targeting neglected tropical diseases, antimicrobial‐resistant infections caused by the ESKAPE pathogens, and their possible application as herbicides. We identify the likely equivalent allosteric pocket in these systems and perform a structure and sequence‐based analysis of the residues comprising it, providing a rationale for continued exploration of this compound series as both specific and broad‐range inhibitors. The review concludes by emphasizing the importance of continued research into ATCase inhibitors, given their potential broad applicability in treating diverse diseases to enhance both human health and agricultural practices.

## Graphical abstract

Pyrimidine biosynthesis is a pivotal process shared by both eukaryotes and prokaryotes. This review examines the applications and advantages of a series of inhibitors specifically designed to target aspartate transcarbamylase within the de novo biosynthetic pathway of the malarial parasite. Members of this small molecule series have also shown inhibitory activity against a range of other organisms, suggesting a common scaffold may provide solutions to a number of biomedical challenges

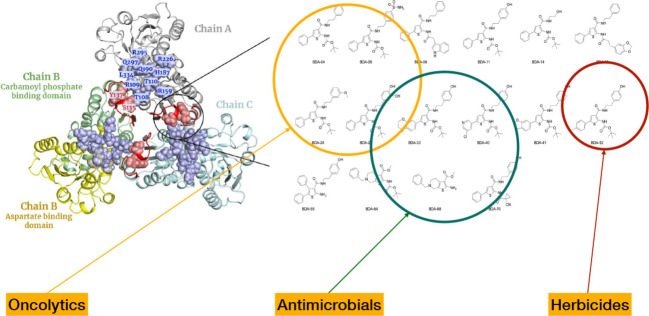

## 1 Introduction

Pyrimidine nucleotides are fundamental to the cell, serving as activated precursors of DNA and RNA, second messengers, precursors of CDP‐diacylglycerol phosphoglyceride for the assembly of biological membranes, and UDP‐sugars for protein glycosylation and glycogen synthesis. These essential molecules are obtained via two routes: either salvage pathway or *de novo* biosynthesis and, depending on cell type and developmental stage, either the salvage pathways or the d*e novo* pathway contribute to the available pool. In general, the activity of the salvage pathway is sufficient to obtain pyrimidines in somatic cells.^[1]^ However, in proliferating cells the *de novo* pathway is indispensable as the demand for nucleic acid precursors and other cellular components increases. Additionally, some pathogens lack a functional salvage pathway. Consequently, blocking the pyrimidine *de novo* biosynthesis becomes a potential treatment for various diseases such as malaria,^[2]^ tuberculosis,^[3]^ oncology,^[4]^ and multiple autoimmune disorders.^[5]^

While the pyrimidine salvage reuses degraded pyrimidine bases (uracil, cytosine, and thymine), the pyrimidine *de novo* pathway synthesizes nucleotides from scratch and is coordinated by six enzymes: the formation of carbamoyl phosphate (CP) is catalyzed by carbamoyl phosphate synthetase II (CPSase II). Carbamoyl phosphate and aspartate are then used in the second step to form N‐carbamoyl aspartate, catalyzed by aspartate transcarbamoylase (ATCase). Remarkably, ATCase is considered the first committed enzyme in this pathway because this reaction is the first irreversible step specific to pyrimidine nucleotide production. Dihydroorotase (DHOase)^[6]^ and dihydroorotate dehydrogenase (DHODHase)^[7]^ are the third and fourth enzymes in the pathway, respectively. DHOase catalyzes the conversion of N‐carbamoyl aspartate into dihydroorotate (DHO), which is then oxidized to orotate by DHODHase. Orotate is then converted into orotidine monophosphate (OMPase) through two distinct enzymatic reactions catalyzed by the fifth and sixth enzymes of the pathway. First, orotate phosphoribosyltransferase (OPRTase) attaches a ribose phosphate group to orotate, producing orotidine monophosphate. Then, orotidine 5′‐monophosphate decarboxylase (OMPDCase) catalyzes the removal of a carboxyl group, resulting in the formation of the final pathway product, uridine monophosphate (UMP). It has been recently shown that in some bacteria and plants, UMP provides negative feedback towards ATCase allosterically.^[8]^ In eukaryotes (excluding plants) ATCase, along with CPSase and DHOase, form a multienzyme complex known as CAD. On the contrary, in most prokaryotes and plants, the enzymes show a lower degree of coordination and are proposed to work independently.

**Figure 1:**
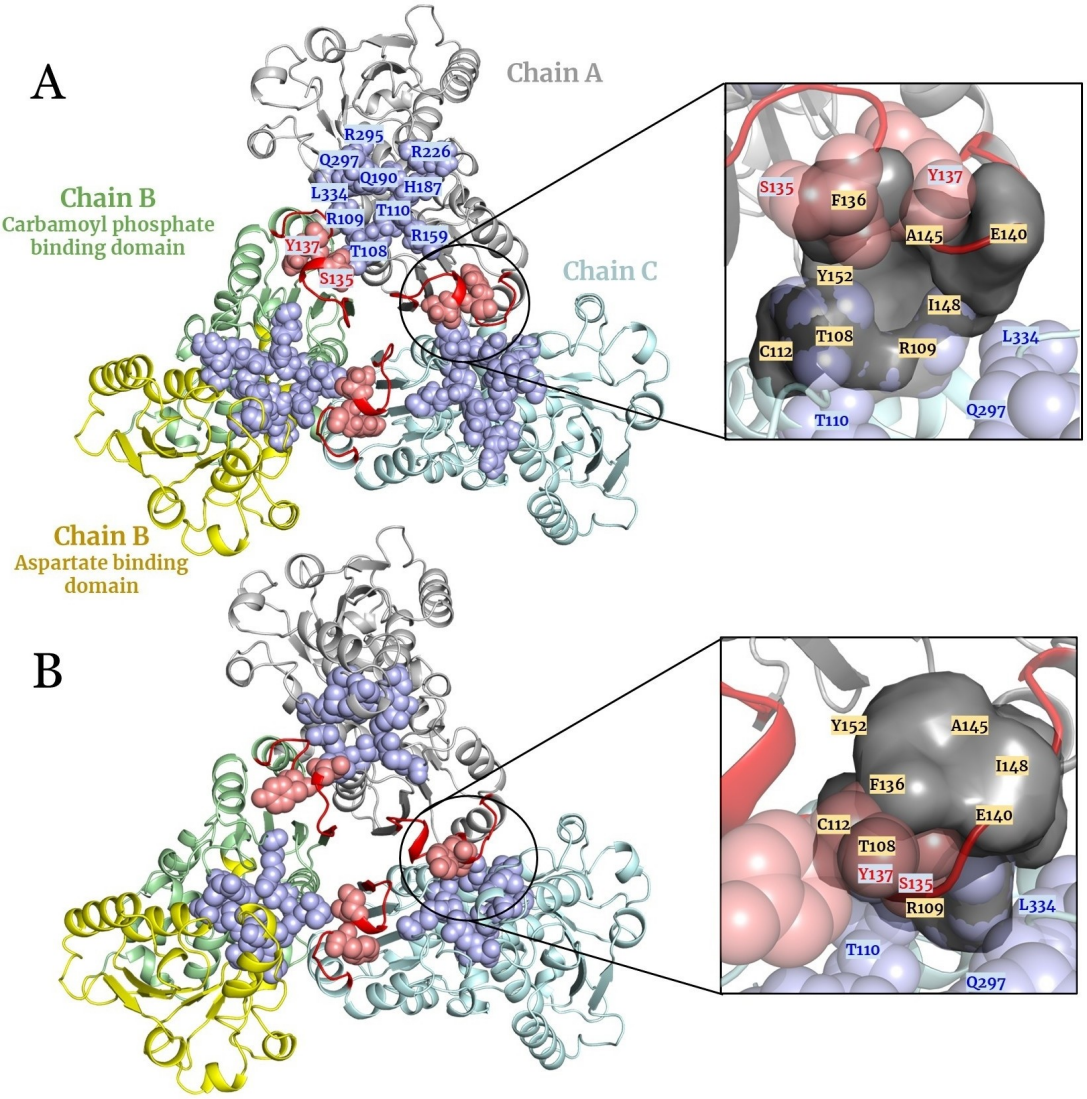
(A) Three‐dimensional (3D) crystal structure of *Pf*ATCase in T state (PDB ID: 7ZP2) and modelled (B) R state (with z‐DOPE score=‐2.12; modelled to fill missing residues of PDB ID: 5ILN) showing the important regions of the enzyme. Chain A, B, and C are shown in grey, pale green and yellow, and pale cyan, respectively. Each monomer has a carbamoyl phosphate (CP) binding domain and aspartate binding domain represented in chain B as pale green and yellow, respectively. The active site residues T108, R109, T110, S135, Y137, R159, H187, Q190, R226, R295, Q297, L334 are shown in spheres and labelled. Each active site contains residues from two adjacent chains. The light blue spheres indicate active site residues located in the main chain, while the light red spheres indicate the active site residues from the adjacent chain. The 120 loop (128 ‐ 142) is shown in red. In each state, the allosteric pocket is zoomed out and shown as a grey surface, and its residues (T108, R109, C112, F136, E140, A145, I148, Y152) labelled in yellow boxes.

Much research has been focused on different enzymes of the *de novo* pathway. Since ATCase is the limiting step and first committed enzyme (as well as being well‐characterized both structurally and biochemically), recent publications have highlighted the potential of developing compounds that target ATCase as novel treatment avenues for various biomedical needs. ATCase is functional as a homotrimer, with three independent active sites located at the interface between each monomer (Figure 1). Structural studies have shown that ATCase has a T (tense) state and an R (relaxed) state, with the binding of substrates allowing ATCase to transition from the low affinity and activity T state into the high affinity and activity R state.^[9]^ The reaction is initiated by the binding of CP to ATCase, followed by a conformational change in the 120s loop to create the binding pocket for aspartate^[10]^ (Figure 1).

Initial efforts to target ATCase were based on the transition state mimetic N‐phosphonacetyl‐L‐aspartate (PALA) ‐ an analog of the transition state product‐ developed by Collins and Stark in 1971.^[11]^ While reports indicated that PALA has shown strong affinity and potency against ATCase from both *Escherichia coli* and humans, PALA failed as an oncolytic as it showed insufficient anti‐tumor efficacy, potential toxicity as well as resistance due to up‐regulation of the pyrimidine salvage pathway in clinical trials.[^12^, ^13^] There have been efforts to develop PALA analogs to improve efficacy, reduce toxicity, or overcome resistance mechanisms. However, most of these derivatives have not progressed beyond preclinical studies.^[14]^ An additional rationale for the failure of PALA as an oncolytic is also provided by recent data from our lab, in which a triphasic inhibition curve can be seen for isolated human ATCase.^[15]^ While initial inhibition (to ~66 % activity) is clearly present at low PALA concentrations, complete inhibition is only seen at significantly higher concentrations (>10uM). While the samples for these experiments are non‐native (ie. the isolated ATCase domain, rather than the CAD assembly) this lack of complete inhibition may provide a rationale for both the lack of success of PALA as an oncolytic in clinical trials and the rapid development of resistance mechanisms, as this data suggests complete inhibition of ATCase activity would only be available at significantly increased PALA. The initial success of PALA as an ATCase inhibitor in *in vitro* enzymatic and cell‐based studies does, however, provide an argument that an allosteric ATCase inhibitor able to provide full inhibition at low concentration would provide a strong opportunity to inhibit the *de novo* pyrimidine biosynthesis.

**Figure 2:**
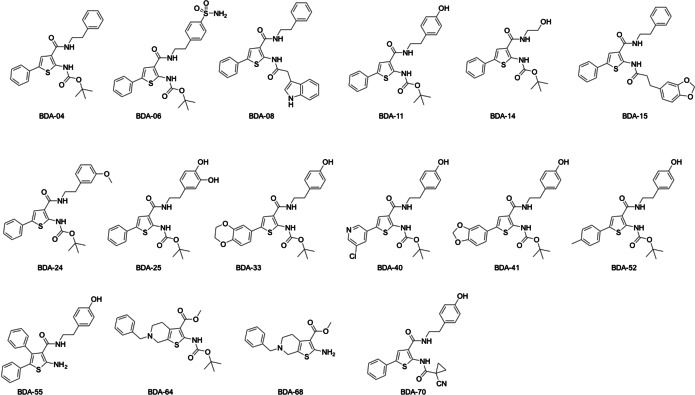
The chemical structures of members of the BDA series described in this manuscript.

To address this gap, we recently performed a fragment screening to identify *P falciparum* ATCase (*Pf*ATCase) inhibitors, resulting in the creation of a compound library of 70 compounds designed to target ATCase activity, allowing the discovery of a new allosteric pocket near the substrate binding sites at the interface of the Aspartate and CP binding domains (Figure 1). As expected, members of this library (hereafter the BDA series (Figure 2)) were also shown to inhibit human and *M tuberculosis* ATCase (*Hs*ATCase and *Mtb*ATCase). A significant advantage of the BDA library is its construction using multicomponent reaction (MCR) chemistry, which allows rapid exploration of chemical space around a single scaffold.^[16]^ Below, we summarize the use of the current library and highlight the potential of using the library as the starting point for the leverage of this single scaffold as a mechanism to generate small molecular weights leads as treatments for further pressing human diseases.

## 2 Demonstrated Interspecies Applications of ATCase Inhibition by the BDA Series

**Figure 3:**
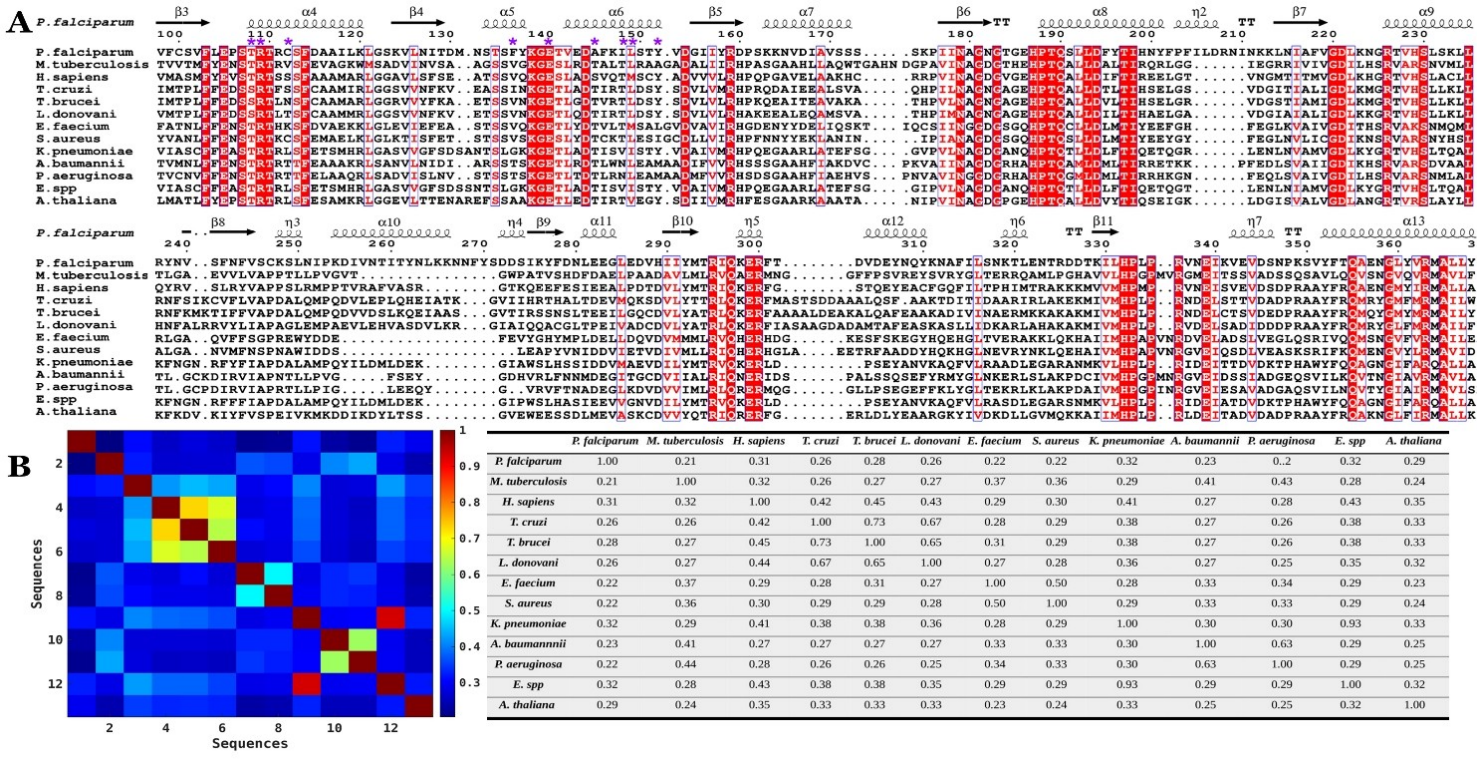
Multiple sequence alignment and sequence identity analysis of the ATCase enzymes from various species. A. Multiple sequence alignment of the ATCase enzymes from *Plasmodium falciparum* (O15804), *Mycobacterium tuberculosis* (P9WIT7), *Homo sapiens* (P27708), *Trypanosoma cruzi* (Q9UAH8), *Trypanosoma brucei* (Q57 U84), *Leishmania donovani* (H9D0Z4), *Enterococcus faecium* (A0 A132PAD8), *Staphylococcus aureus* (A0 A0D1HIF4), *Klebsiella pneumoniae* (A0 A060VJE1), *Acinetobacter baumannii* (A0 A009IHK5), *Pseudomonas aeruginosa* (A0 A069QI11), *Enterobacter spp* (A0 A329H1R9), and *Arabidopsis thaliana* (P49077) was performed using Promals3D[^86^, ^87^] and visualized with ESPript 3.0.^[87]^ The blue boxes show conserved residues with similar physicochemical properties. Highly conserved residues are highlighted in red and conserved residues with residue replacements are shown in red. The allosteric pocket residues are shown as purple asterisks. The header shows the secondary structure associated with each residue where squiggles represent α‐helices, TT represent strict β‐turns, and the arrows represent β‐sheets. B. Heatmap and table show pairwise sequence identities of the ATCases (calculated using an in‐house Python script) on a scale from 0 (0 %) to 1 (100 %) with colors ranging from blue to brown. A sequence identity of 1 is presented in brown, while a sequence identity of 0 is shown in blue. The sequence numbers in the heatmap correspond to the order shown in the table.

**Figure 4:**
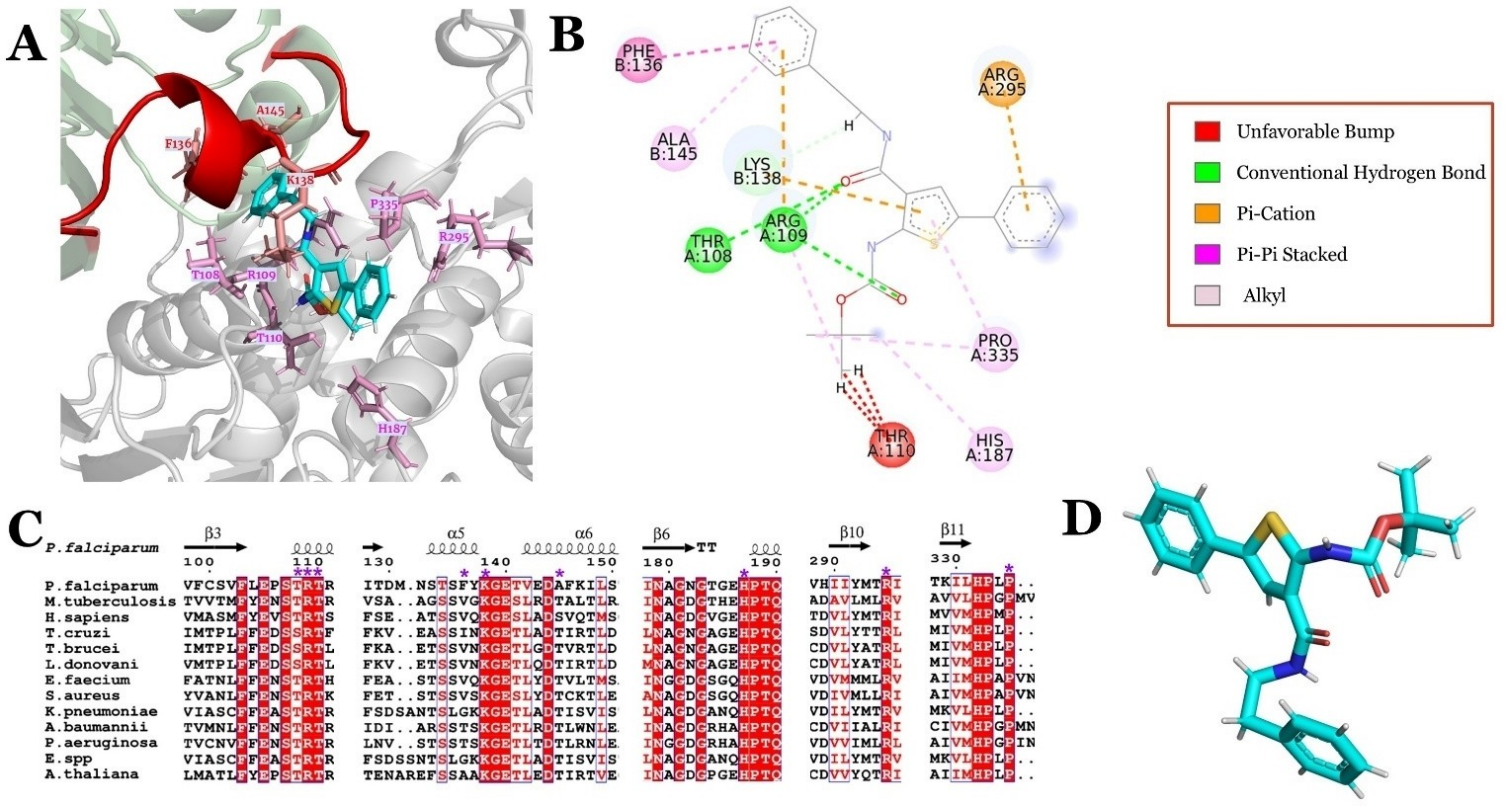
A. Binding of BDA‐04 to the allosteric pocket of PfATCase (PDB ID: 7ZP2) with key interacting residues. Chain A and Chain B are depicted as gray and green cartoons, respectively, with the 120 loop highlighted in red. The interacting residues are represented as stick models. B. Type of interactions between BDA‐04 and allosteric pocket of *Pf*ATCase. C. A zoomed‐in section of the MSA from Figure 3 highlights the interacting residues across various ATCases. The purple asterisks mark the residues that interact with the BDA‐04 compound. D. Structure of BDA‐04.

**Figure 5:**
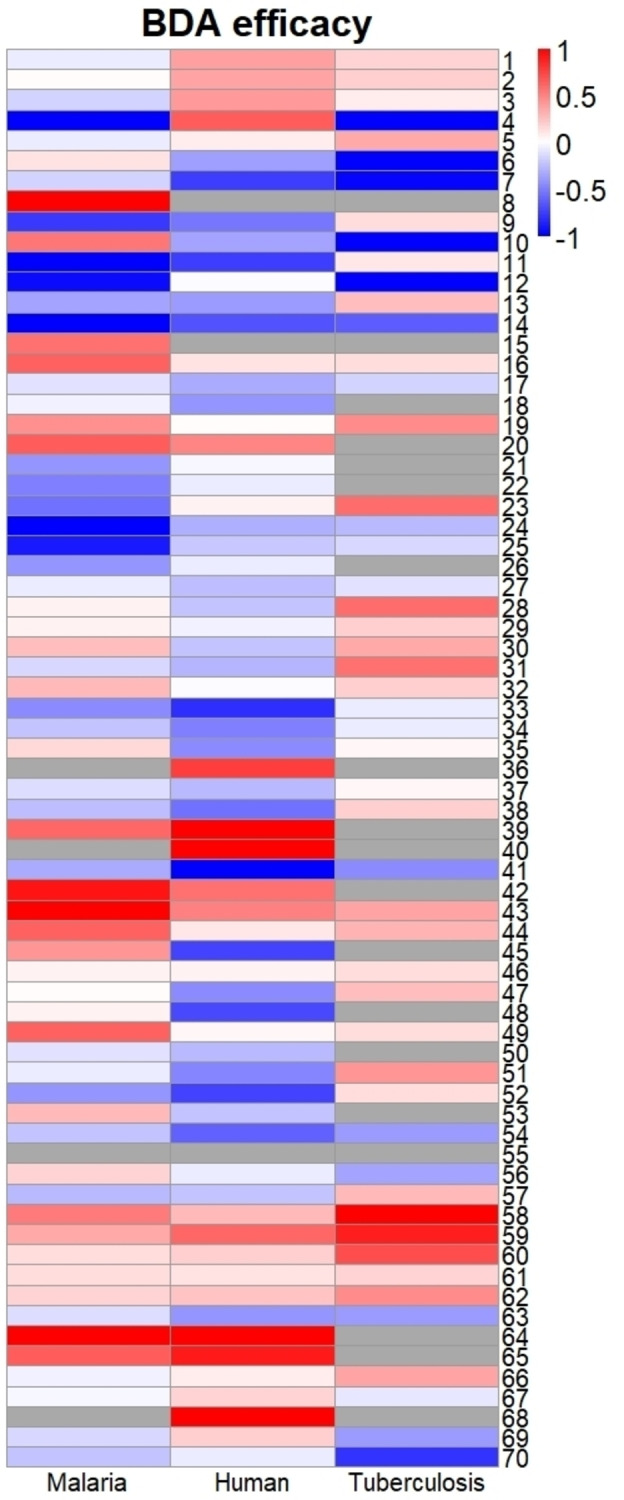
The BDA‐series contains both broad‐range and species‐specific inhibitors as shown by a comparison of BDA series efficacy in malaria, human and tuberculosis ATCases. In this visualisation, the average *in vitro* IC_50_ value for BDA family members within the indicated species was used as a baseline. Variations from this average were calculated as the logarithm of the ratio between the individual value and the species average and coloured as indicated. Data from.[^2^, ^3^, ^15^] The figure demonstrates that some members of the BDA family possess broad‐range inhibitors as they perform well against multiple species (eg. BDA‐14), whereas others are top performers for individual species (eg. BDA‐45). Grey values indicate experiments that did not yield a measurable IC_50_ and are excluded from the calculation.

As Figure 3 shows, ATCase is conserved across different species with the substrate binding sites for CP and aspartate showing a high identity and similarity, while the allosteric site exhibits greater variability. Amongst the 70 compounds of the BDA series, the *in vitro* IC_50_ values against *Pf*ATCase ranged from double‐digit nM to μM. The most potent inhibitor was BDA‐04 with an IC_50_ of 77.2 nM and the co‐structure of this compound with *Pf*ATCase (PDB: 7ZP2) illustrated that BDA‐04 binds allosterically on *Pf*ATC (Figure 4), stabilizing the enzyme in an inactive conformation by locking the 120s loop in a position that blocks the CP binding site and blocking the transition of *Pf*ATCase between the R and T states. Interestingly, the electron density for the bound inhibitor is not equally present in each of the three active sites, suggesting a degree of co‐operativity between the distinct active sites of this ATCase, whereby blocking the R/T state transition of a single monomer inhibits the trimeric holoenzyme. After showing successful inhibition of blood‐stage malarial cultures of the more potent *Pf*ATCase inhibitors, we hypothesized that the same compound library may also contain inhibitors of the ATCase of M tuberculosis (Mtb).Following the screening of the 70‐compound BDA library in an *in vitro Mtb*ATCase enzymatic assay, several compounds showed promising results in the single digit μM range. The most potent inhibitors were analyzed in cell culture experiments for their potential to inhibit *Mtb*. Within these experiments, BDA‐06 was identified as the best‐performing compound with an IC_50_ value of 1.4 μM and MIC_50_ value of 4.0 μM. In addition, host cell cytotoxicity assays also supported the reliance of proliferating cells on the *de novo* pyrimidine biosynthesis pathway. In the interim, we also screened the BDA series for inhibition of *Hs*ATCase through a similar enzymatic assay. The results again supported our hypothesis: nearly all compounds showed inhibition, with several demonstrating low nanomolar inhibition. Notably, BDA‐11, 33, 41, and 52 exhibited strong inhibition of *Hs*ATCase, with IC_50_ values of 116 nM, 101 nM, 29 nM, and 119 nM, respectively. Interestingly, unlike *Pf*ATCase and *Mtb*ATCase, over 50 % of the BDA series compounds displayed an *in vitro* IC_50_ in the nanomolar range, indicating high selectivity of these compounds between different species. One of the most effective molecules, BDA‐41, displayed an anti‐proliferation effect on U2O osteoblastoma cells by promoting cell cycle arrest in the G0/G1 phase, suggesting that further optimization could yield an anti‐cancer cancer agent. Among the three homologous ATCases described above, BDA‐14 achieved good inhibition in all cases tested while BDA‐08, BDA‐15, BDA‐40, BDA‐55, BDA‐64, and BDA‐68 seem to be poor inhibitors against all ATCases tested, providing further insights for the compound optimization (Figure 5). Although ATCases share significant sequence similarity, the residues within the potential BDA binding site exhibit variations (Figures 3 & 4A−C). These differences provide both a rationale for the observed variation in IC50 values and an opportunity to develop species‐specific compounds. The potential to exploit BDA series targeting the novel allosteric pocket to generate species‐specific lead molecules is exemplified by the data that shows that BDA‐24 and BDA‐25 are more specific to malarial ATCase, while BDA‐41 is more specific to human ATCase and BDA‐06 & BDA‐70 are more specific to tuberculosis ATCase.

In the following section we will expand on the potential and need to exploit variations around a single scaffold to generate species‐specific leads for ATCase inhibitors.

### 2.1 Malaria

Over the past decades, significant efforts have been made to control malaria, but it still remains a global challenge. According to the World Health Organization (WHO), it is estimated that there were 249 million cases and 608,000 deaths in 85 countries in 2022. In addition, there was an increase of 5 million cases in 2021 associated with the impact of the COVID‐19 pandemic.^[17]^ There are 5 types of Plasmodium parasites that can cause human malaria, of which *P falciparum* and *P vivax* account for more than 95 % of cases. While Artemisinin‐based combination therapies (ACTs) are the first‐line treatment, drug resistance is emerging. In 2021 the WHO approved the RTS,S/AS01 malaria vaccine, which is a significant milestone in the antimalarial campaign.^[18]^ Two years later, the R21/Matrix‐M vaccine was approved for use in children in endemic areas, with 75 % efficacy reported in February 2024.^[19]^ Although the two vaccines are shown to reduce malaria incidence rate by more than half during the first year after injection, malaria drugs are still needed for active infection. Additionally, malaria parasites are highly adaptable, and new mutations can arise to evade both immune responses induced by vaccines and the effects of existing drugs. This situation is significantly exacerbated, as vaccine‐based elimination of the malarial parasite through vaccination would require unprecedented global efforts and addressing the potential for zoonotic transfer of plasmodial species between animals and man cannot be addressed by human vaccination alone.^[20]^ Therefore, continuous drug development for malaria is still essential. As the enzyme supporting the second step of the *de novo* pyrimidine biosynthesis pathway, ATCase has been demonstrated to be a promising target for antimalarial development.[^2^, ^21^]

In our fragment‐based approach to developing inhibitors of the malarial ATCase (*Pf*ATCase),^[2]^ several complex structures were generated from the *Pf*ATCase “T” state (PDB: 7ZCZ, 7ZEA, 7ZGS and 7ZHI), combined with series of *in vitro* experiments, which demonstrated the presence of a new allosteric pocket near the traditional substrate binding site (Figures 1 & 4A). The compound 2,3‐napthalediol was shown to inhibit in a substrate‐independent manner, with the associated structures demonstrating that the allosteric binding pocket is composed of both the Aspartate and CP domains (Figure 1).^[22]^ The proposed mode of action is that the compound prevents the “T” to “R” state transition by stabilizing the inactive state. As detailed above, a series of compounds (the BDA series) were designed from these data and evaluated as potent noncompetitive inhibitors through *in vitro* enzymatic and kinetic assays,^[2]^ with the most potent inhibitor, BDA‐04 (Figure 4D), displaying an IC_50_ of 77.2 nM and an EC_50_ of 2.4 μM (PDB: 7ZP2). This structure, as well as BDA‐14:*Pf*ATCase (PDB: 7ZID), illustrated that the BDA series partially occupied the CP binding domain, and further supported the proposed allosteric mechanism and mode of action (Figure 1& 5 A). The most active BDA compounds were also shown to possess good cytotoxicity and demonstrated strong performance in the inhibition of blood‐stage malaria culture.^[2]^

### 2.2 Tuberculosis

Tuberculosis (TB) is mainly caused by *Mtb* and spreads between people through the air, typically affecting the lungs of the host. The pathogenesis consists of two stages, an initial latent stage and a second active stage. In 1993, TB was announced to be a global disease by the WHO, who have published a global TB report every year since 1997. Despite decades of research, there are still 1.6 million deaths around the world annually.^[23]^ The most widely used vaccine was introduced more than 70 years ago, with the development of the bacillus Calmette‐Guérin against tuberculosis.^[24]^ However, the rise of multi‐drug resistant and extensively drug‐resistant strains significantly increases the threat associated with TB, further complicated by the increased risks of co‐infection with human immunodeficiency virus (HIV).^[25]^ Similarly to malaria, there is a continuous need for new targets for the development of new inhibitors against TB. Enzymes implicated in cell wall biosynthesis have already proven to be an excellent drug development target since their specific characteristics, for example the biosynthesis of mycolic acid, arabinan and lipoarabinomannan.[^26^, ^27^, ^28^] However, the development of new targets supporting fundamental biochemical functions and/or involved in essential cellular metabolic pathways should also be evaluated for drug development.

The pyrimidine biosynthesis pathway was long thought to be one of the essential nucleotide metabolism pathways in Actinobacteria, including *Mtb*.^[29]^ Although the details of latent TB stage metabolism are not fully understood, Boshoff and colleagues demonstrated the importance of nucleotide synthesis in this stage.^[30]^ Since the gene sequence of *Mtb* H37Rv strain was fully characterized, the gene encoding in the *de novo* pyrimidine biosynthesis map was identified,^[31]^ and inhibitors of the targeted nucleotide biosynthesis enzyme could be a promising agent against both the first latent TB stage and the second active TB stage. Pyrimidine‐2(1H)‐one‐based inhibitors of orotate phosphoribosyltransferase (OPTase), which catalyzes the fifth reaction in the *de novo* pyrimidine biosynthesis pathway, were synthesized and investigated as potential drug leads,^[32]^ supported by the of crystal structure characterization of *Mtb*OPRTase,^[33]^ making it another attractive inhibitor in pyrimidine biosynthesis. This further supports the essential role of pyrimidine biosynthesis in *Mtb*. Nevertheless, this inhibitor is only useful for in vitro inhibitory activity, as it is not specific to the *Mtb*OPRTase. Although kinetic analysis and *in silico* docking results indicated that BDA‐06 may inhibit *Mtb*ATCase through a similar allosteric pocket as seen in *Pf*ATCase, the crystal structure of *Mtb*ATCase in complex with BDA‐06 is still required to confirm the proposed binding mode.

### 2.3 Oncology

Cancer has been an intense research topic in recent decades and many targets are involved in *de novo* pyrimidine biosynthesis. For example, Zhang found that combining targeting DHODHase and the anti‐apoptotic BCL2 L1 significantly enhances the effectiveness of treatment in pancreatic cancer cells.^[34]^ The relative reliance on the different pyrimidine synthesis pathways differs between normal and neoplastic cells, due to the higher demand for nucleotides in tumor cells compared to normal cells as generally, the salvage pathway suffices for the needs of normal cells.^[1]^ However, in proliferating cells, including tumor cells, the demand for nucleic acid precursors significantly increases, making the de novo synthesis pathway indispensable.[^35^, ^36^] In this case, inhibitors of enzymes in the *de novo* pyrimidine biosynthesis pathway have been demonstrated to be potential drug candidates for treatment. This is supported by the in silico discovery of inhibitors (YD‐19 and YD‐21, in vitro KD of 13 and 18 μM, respectively) that target the human ATCase and show an impact on tumour growth in mouse xenograph models.^[37]^

Unlike the substrate competitive inhibition mechanism shown by PALA against *Hs*ATCase, the BDA series compounds, previously described as anti‐malarial and anti‐tubercular candidates, were shown to inhibit *Hs*ATCase through a substrate noncompetitive mechanism. The allosteric pocket required to support this mechanism may also exist in humans, as evidenced by the kinetic assay.^[15]^ Although the structure of the flexible loop (equivalent to the 120s loop of *Pf*ATCase) was absent in the available structure of the T state of *Hs*ATCase, the key residues of the malarial structures (7ZP2 and 5ILQ) are fully conserved. This suggests that a similar function can be assigned to the 120s loop in HsATCase, which would be stabilized by the binding of the BDA series, and prevent the binding of CP (Figure 4). Therefore, we conclude that the BDA series may be a propitious anti‐tumor candidate.

## 3 Potential Future Applications of ATCase Inhibition by the BDA Series

### 3.1 Neglected Tropical Diseases

Neglected tropical diseases (NTDs) encompass a group of diverse infections that primarily affect populations living in poverty in tropical and subtropical regions. These diseases, though preventable and treatable, receive insufficient attention from global health initiatives and research efforts, exacerbating their burden on affected communities. Among the NTDs, parasitic diseases caused by protozoan pathogens stand out due to their debilitating effects and complex transmission cycles. Three of the most significant protozoan NTDs are African trypanosomiasis (caused by *Trypanosoma brucei*), Chagas disease (also known as American sleeping sickness, caused by *Trypanosoma cruzi*), and Leishmaniasis (caused by *Leishmania* parasites). These diseases not only contribute to high mortality and morbidity rates, but also perpetuate cycles of poverty in endemic regions. Due to the significant adverse effects of current anti‐trypanosomiatid drugs and the increase in drug resistance,^[38]^ new drugs and treatments are urgently needed. A current focus is on lipidomic metabolism pathways in trypanosomiasis, which have provided a foundation for new drug development.^[39]^ However, the nucleic acid/base synthesis pathway could also be considered as a potential drug target due to its essential role in most living organisms, including trypanosomiasis, especially during the proliferating stage in the host.^[40]^

#### 3.1.1 Sleeping Sickness

It has been shown by Hammond and Gutteridge that both the bloodstream form and intracellular form of *T. cruzi* are dependent on the *de novo* pyrimidine pathway to proliferate, although they potentially have the salvage pathway .^[41]^ Hashimoto *et al*. also revealed that the knockdown of the CPSase II gene significantly suppressed the growth of the intracellular replicating form of *T. cruzi*.^[42]^ Thus, as the key enzyme of the *de novo* pyrimidine biosynthesis pathway, ATCase can also be considered as a potential target for the development of new inhibitors for Chagas disease.

Following the preliminary X‐ray analysis of *T. cruzi* ATCase (*Tc*ATCase),^[43]^ the crystal structure of *Tc*ATCase, both for apo‐ and carbamoyl aspartate complex, were reported (PDB: 6JKQ, 6JL4) providing T‐ and R‐state structures similar to those observed in *Pf*ATCase. By extrapolating binding site and allosteric pocket residue information (Figures 1 & 3) and incorporating insights from the BDA‐04‐*Pf*ATCase complex analysis into a residue‐level assessment (Figure 4C), we hypothesize that a similar allosteric pocket also exists in *T. cruzi*. Following promising results of inhibition by the BDA series against *Pf*ATCase, *Mtb*ATCase and *Hs*ATCase, we assessed this series against *Tc*ATCase and BDA‐41 provided promising results with an IC_50_ in the low nanomolar range (manuscript in preparation).

Although *T. cruzi* and *T. brucei* are both protozoan trypanosomes, Ali *et al*. have shown that *T. brucei* utilizes both the pyrimidine salvage and *de novo* biosynthesis pathways to meet nucleotide requirements.^[44]^ However, ATCase may still be a therapeutic target for African sleeping sickness when host‐derived pyrimidines are insufficient.

#### 3.1.2 Leishmaniasis

Leishmaniasis is caused by *Leishmania* parasites, from more than 20 species, which are vector‐born and spread by the female phlebotomine sandfly only one‐quarter the size of a mosquito. This disease consists of cutaneous leishmaniasis (CL), visceral leishmaniasis (VL) and mucocutaneous leishmaniasis (MCL), spreading across more than one hundred countries around the world, including Africa, the Americas, Europe, Asia, and infecting both mankind and animals. There are still more than 1 million new cases of CL reported annually.

The purine salvage pathway was identified to be a promising drug target since leishmania‐causing species lack a purine *de novo* synthesis pathway but retain active salvage pathways.^[45]^ In contrast, leishmania has both *de novo* pyrimidine biosynthesis and salvage pathways, which were initially not considered to be potential drug targets.[^46^, ^47^] However, due to the differences in functional proteins and regulation between leishmania and humans, the pyrimidine biosynthesis pathway has been reconsidered and proposed to be another encouraging target. While knockouts of the *de novo* synthesis pathway do not fully attenuate leishmaniasis, rather partly reducing infection both *in vivo* and *in vitro*, equally knockouts of the salvage pathway do not reduce leishmania's effectiveness.^[48]^ This leads to the potential of a combinatorial approach of inhibiting the *de novo* and salvage biosynthesis pathways. In this scenario, the ATCase from *Leishmania* remains a target for research. 3D conservation of *Leishmania* ATCase (PDB: 7K46) with PfATCase (PDB: 7ZP2) is less evident at the sequence level, with a sequence identity of 26 % (Figure 3B). However, most of the *Pf*ATCase residues interacting with BDA‐04 compound are also conserved in *Leishmania* ATCase (Figure 4C), suggesting the possibility of a similar allosteric inhibition mechanism in *Leishmania* ATCase (Figure 4B). Nonetheless, *in vitro* inhibition by the BDA library is still to be assessed.

### 3.2 Antimicrobial Resistance: ESKAPE Pathogens

With the appearance and increase of antibiotic resistance and multi‐drug resistance of bacteria, there are small group that are the major cause of nosocomial infection^[49]^ consisting of six bacterial pathogens: *Enterococcus faecium*, *Staphylococcus aureus*, *Klebsiella pneumoniae*, *Acinetobacter bacumannii*, *Pseudomonas aeruginosa*, and *Enterobacter spp*, ‐ collectively named ESKAPE (sometimes extended to ESKAPEE with the addition of *Escherichia coli* ^[50]^). These pathogens are already a threat to public health, due to the expensive cost or ineffective therapy.^[51]^ In 2017, the first 12 catalogues of antibiotic‐resistant bacteria were published by the World Health Organization (WHO), which were divided into three groups according to the threat to mankind: Critical, high, and medium. are small group that are the major cause of nosocomial infection.^[49]^ Of these three levels, four of the ESKAPE organisms: *Kelbsiella*, *A baumannii*, *P. aeruginosa* and *Enterobacteriaceae* were placed into the ”critical” group. The other two, *E. faecium* and *S. aureus*, are present in the ”high” group. However, this ”high” group displays an increase in disease occurrence and drug resistance continually.^[52]^

In addition to the direct chemical treatments of antimicrobial infections, other methods were considered, such as bacteriophage therapy,^[53]^ antimicrobial peptides,^[54]^ photodynamic light^[55]^ and silver nanoparticles.^[56]^ However, one of the most common reasons for the increase in microbial resistance is the continual overuse and misuse of antibiotics. Within developed and/or developing antimicrobial resistance strains, the impact of current treatments is continually declining, indicating that future therapy methods and new antimicrobial pathogens combating ESKAPEE should be urgently and continuously investigated. Essential metabolic pathways are clearly in the eyes of researchers – including nucleotide biosynthesis pathways, not only because of their central role in the function of survival and proliferation but also due to the strong link between the production of virulence factors and nucleotide biosynthesis.^[57]^ Although the mechanism and efficacy still need to be elaborated, the pyrimidine *de novo* biosynthesis pathway and its regulators have been demonstrated as a potential targets for the development of new antibiotics.^[57]^ PALA, a high‐efficacy inhibitor of ATCase, was shown to enhance clearance of methicillin‐resistant bacteria with an indirect inhibition mechanism in human skin explants in both *S. aureus* and *A. baumannii* ^[54]^ infection models, clearly showing the potential for inhibition of *de novo* pyrimidine biosynthesis.

#### 3.2.1 Staphylococcus Aureus

*S. Aureus* belongs to the Gram‐positive, cocci‐shaped bacterium and is one of the most common causes of skin infections from children to adults,^[58]^ and also has been identified in osteoarticular infection^[59]^ . This bacterium can cause a range of illnesses, for instance, pneumonia,^[60]^ bacteremia,^[61]^ ocular infections,^[62]^ and endocarditis.^[63]^ The most common treatments are vancomycin, methicillin and similar antibodies. However, vancomycin‐resistant and methicillin‐resistant *S. aureus* (MRSA) strains are emerging.^[64]^ Besides, the treatment becomes less effective because *S. aureus* strains are able to secrete a large variety of virulence factors that are encoded on mobile genetic elements, and one of the functions is to help it evade the host immune system. Other factors have additional potent toxic functions, amongst which the toxic shock syndrome toxin (TSST) was proven to cause a potentially serious disease. Around 25 % of *S. aureus* strains secrete TSST‐1.[^65^, ^66^] In addition to the problems in developed/developing drug resistance, these features increase the suffering of infected patients. Thus, novel drug targets are urgently needed.

It has been demonstrated that the pyrimidine biosynthesis pathway is related to the colonization of *S. aureus*, according to colonization experiments in mice strains with pyr operon mutations.^[67]^ The pyr operon regulates pyrimidine biosynthesis, which greatly increased interest in potential targets within the pyrimidine biosynthesis pathway. The oxidate catalyzation of DHO to orotate step has been identified as being associated with the respiratory chain from *S. aureus*,^[68]^ meaning dihydroorotase (DHOase) as the third enzyme of the *de novo* pyrimidine biosynthesis pathway was suggested as a potential antimicrobial target.^[69]^ Recently, a 1‐benzylpiperidine‐4‐ol scaffold was considered to be a novel compound against DHOase of *S. aureus* and this scaffold displayed a ~10 times higher K_D_ value than its substrate.^[70]^ In this case, we hypothesize that the second catalytic enzyme, ATCase of *S. aureus* (*Sa*ATCase), also could be a potential drug target. Thus, the BDA library designed against *Pf*ATCase will be assessed against *Sa*ATCase in the near future.

#### 3.2.2 Acinetobacter Baumannii

*Acinetobacter baumannii* mainly causes bloodstream infections and (respiratory tract) ventilator‐associated pneumonia, as well as infections with secondary meningitis and endocarditis.^[71]^ Along with *S. aureus*, it was classified as the most critical pathogen by the WHO, and, when infection occurs in the presence of other injuries or diseases, leads to an increase in mortality rate and a serious threat to public healthcare. Since it possesses a highly plastic genome, which can mutate to adapt to diverse environments, *A. baumannii* has been found to exhibit nearly all kinds of antimicrobial resistance, for instance, cephalosporine resistance,^[71]^ carbapenem‐resistance,[^72^, ^73^] polymyxin resistance,^[74]^ and quinolones resistance.^[75]^ Thus, *A. baumannii* was identified as one of the most troublesome pathogens worldwide^[76]^ and the need for the development of new antimicrobials against *A. baumannii* through novel targets has been identified.

The *de novo* pyrimidine biosynthesis pathway is essential for *A. baumannii* survival and proliferation, as genetic studies have shown that *A. baumannii* lacks the complete enzyme pathway for the salvage of pyrimidine nucleotides.^[77]^ Therefore, it is reasonable to hypothesize that the bacterium relies on the *de novo* biosynthesis pathway to synthesize pyrimidine nucleotides required for DNA and RNA synthesis. DHODHase – the fourth enzyme of the *de novo* pyrimidine biosynthesis pathway, was reported to be a potential drug target for the treatment of *A. baumannii* infections. A compound known as DMS186 was identified to be a potent candidate against *A. baumannii*, including several resistance strains.^[77]^ This further demonstrates the importance of pyrimidine biosynthesis for this bacteria species, and ATCase as the second step in the pyrimidine biosynthesis pathway could again be proposed as a druggable target.

A similar BDA binding pocket may exist in *Ab*ATCase. While the sequence identity between these two proteins is 23 % (Figure 3B), there is a clear conservation in the 120s loop and allosteric pocket residues (Figures 3A & 4C).

### 3.3 Plants

Aspartate transcarbamoylase is a major enzyme in *de novo* pyrimidine biosynthesis in the genus Arabidopsis, including *Arabidopsis thaliana* (*At*ATCase) (member of the mustard family), located within the plastid. Additionally, the *de novo* pyrimidine biosynthesis process occurs across the compartments of the chloroplast, cytosol and mitochondria.[^8^, ^78^] UMP, the final product of this process, serves as a double‐action inhibitor. On the one hand, UMP regulates CPSase activity, with its inhibitory effect counterbalanced by ornithine, which stimulates *At*ATCase activity and prevents UMP‐induced inhibition in Cucurbita Pepo cell‐free extract.^[79]^ On the other hand, UMP directly binds to and blocks the active site of *At*ATCase in the chloroplast, competing with CP and exerting negative feedback on *At*ATCase activity, further modulating the metabolic pathway.[^8^, ^80^]

In addition to its physiological relevance in pyrimidine biosynthesis and orotic acid pathway regulation,^[79]^ knockdown *A. thaliana* lines have demonstrated the essential role of ATCase in plant growth and the photosynthetic process.^[8]^ Several studies have revealed that treatment with PALA leads to reduced plant biomass, altered leaf and root growth, and chlorosis inhibition in Arabidopsis seedlings.[^78^, ^81^, ^82^] Moreover, in *At*ATCase, PALA can bind sequentially to the active sites of the trimer due to an interaction between active sites ‐ facilitated by a loop folding within the CP binding domain. A mutation of F161 to alanine in *At*ATCase increases the affinity of PALA for ATCase, allowing the simultaneous binding of three PALA molecules and resulting in an *At*ATCase construct unresponsive to UMP.^[8]^ These findings underscore the importance of ATCase in plant growth and suggest inhibiting this enzyme could hinder the growth *of A. thaliana*.

The results of an *in vitro* enzymatic assay from the BDA series against *At*ATCase combined with a kinetic assay, further demonstrated our hypothesis (manuscript in preparation). Efforts will be made to obtain the structure of *At*ATCase with the BDA series to investigate a potential binding mode similar to that observed in the *Pf*ATCase:BDA‐04 complex.

## 4 Summary and Outlook

This review provides a current overview on the potential for ATCase inhibition across a broad range of biomedical needs. Pyrimidine biosynthesis is a fundamental pathway essential for all living organisms, with ATCase catalyzing the second critical step in the de novo biosynthesis process. However, a confounding factor is that the currently available molecules addressing pyrimidine biosynthesis (eg. PALA,^[83]^ DSM265^[84]^) are targeted towards the cognate active sites of the enzymes involved. These sites are highly conserved due to evolutionary pressure, and the active sites are highly conserved, limiting opportunities to develop species‐selective inhibitors. It is noteworthy that resistance to the anti‐malarial DSM265 has already been observed.^[85]^

In contrast, our recently developed series of potent ATCase inhibitors[^2^, ^3^, ^15^] leverage the increased sequence variation in the enzyme allosteric binding site to achieve selectivity. Importantly, while our compound series was initially designed for the malarial enzyme, some inhibitors demonstrated superior efficacy against human or (myco)bacterial CPSase, achieving either species selectivity or pan‐species activity. This versatility underscores ATCase's potential as a drug target for treating diverse diseases, including malaria, tuberculosis, human cancers, neglected tropical diseases, and antimicrobial‐resistant infections. While data is lacking to conclusively demonstrate that all BDAs bind to the same site all tested BDAs share an allosteric mechanism of inhibition and homology modeling indicates that all BDAs examined could locate to the proposed binding site.

These diseases have significantly impacted public health, particularly in under‐resourced regions, where cost‐effective treatments are urgently needed. Developing broad‐range drugs targeting ATCase based on a single scaffold offers an economically viable solution. This approach reduces development costs, streamlines production, and minimizes the need for extensive diagnostic infrastructure by addressing multiple diseases with a single development effort. Simultaneously, computational tools—such as molecular modeling, dynamic simulations, and AI‐driven optimization—can substantially shorten lead development timelines. These advanced techniques enhance efficiency while ensuring the identification of selective and potent inhibitors, accelerating the path to effective treatments.

To further harness ATCase as a therapeutic target, it is critical to identify and develop new chemical entities capable of selectively and potently inhibiting the enzyme. By targeting allosteric sites or exploring protein‐protein interaction interfaces, these novel molecules could provide a pathway to enhanced selectivity, reduced off‐target effects, and greater efficacy across diverse species. This approach not only addresses the challenges of highly conserved active sites but also opens new avenues for innovative drug design.

Clearly, the exploration of ATCase inhibitors holds promise ‐ not only in advancing human healthcare but also in enhancing agricultural practices, especially given the role of this pathway in plant growth and health. However, research into this pathway still needs to be prosecuted, as there is still a large degree of uncertainty regarding individual species′ reliance on the *de novo* or salvage pathways.

## Conflict of Interests

The authors declare no conflict of interest.

## Biographical Information

*Siyao Chen received her MSc in Drug Discovery and Translational Biology from the University of Edinburgh in 2021. She is currently a third‐year PhD student at the University of Groningen, supported by the Chinese Scholarship Council, under the supervision of Matthew Groves. Her research focuses on developing ATC inhibitors as multi‐purpose drugs, including antimalarials and anti‐trypanosomatids*.